# Supplementary material for: Endothelial Jak3 expression enhances pro-hematopoietic angiocrine function in mice
Source: Commun Biol. 2021 Mar 25;4:406. doi: 10.1038/s42003-021-01846-3 (PMC7994450; doi:10.1038/s42003-021-01846-3)
Supplement: Supplementary file 1 — Supplementary Information [file 42003_2021_1846_MOESM1_ESM.pdf]

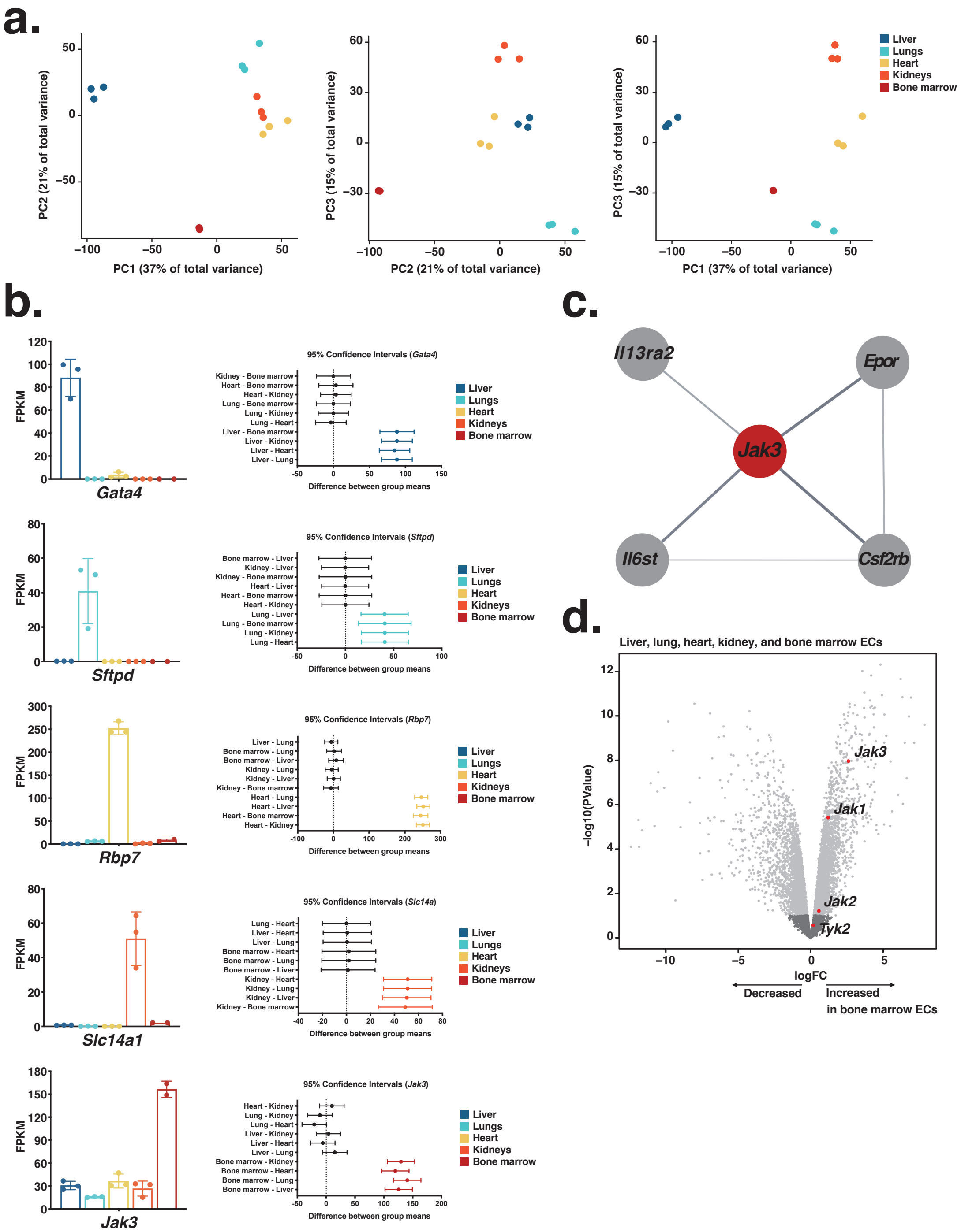

Supplementary Figure 1. Organotypic transcriptomic signatures of endothelial cells (ECs). (a) Principal component analysis (PCA) performed on RNA sequencing (RNA-seq) counts of primary murine endothelial cell populations extracted from the indicated vascular beds where principal component 1 (PC1), PC2, and PC3 are illustrated plotted against each other. (b) Average FPKM from organotypic endothelial transcript expression across vascular beds obtained by RNA-seq (left, n = 2 or 3). Four asterisks (****), three asterisk (***), and two asterisks (**) denote, respectively, p values < 0.0001, < 0.001, and < 0.01 by one-way ANOVA. Each replicate is represented by an individual dot and error bars represent the standard deviation from the mean. Plots of 95% confidence intervals (right) generated by one-way ANOVA comparisons of indicated gene transcripts in every two vascular niches analyzed. (c) Network visualization of known and predicted protein-protein interactions between gene transcripts corresponding to the JAK-STAT signaling pathway following analysis of differentially expressed bone marrow EC gene transcripts against the Kyoto Encyclopedia of Genes and Genomes database[54–56]. (d) Volcano plot illustrating differential expression analysis of RNA-seq corresponding to bone marrow ECs vs. liver, lung, heart, and kidney ECs. Specific gene transcripts are highlighted with enlarged dots in red; respectively, light or dark gray dots indicate gene transcripts differentially expressed above or below cutoffs, $-\log_{10}$(p value) < 1 and $|\log_2 FC| < 2.5$.

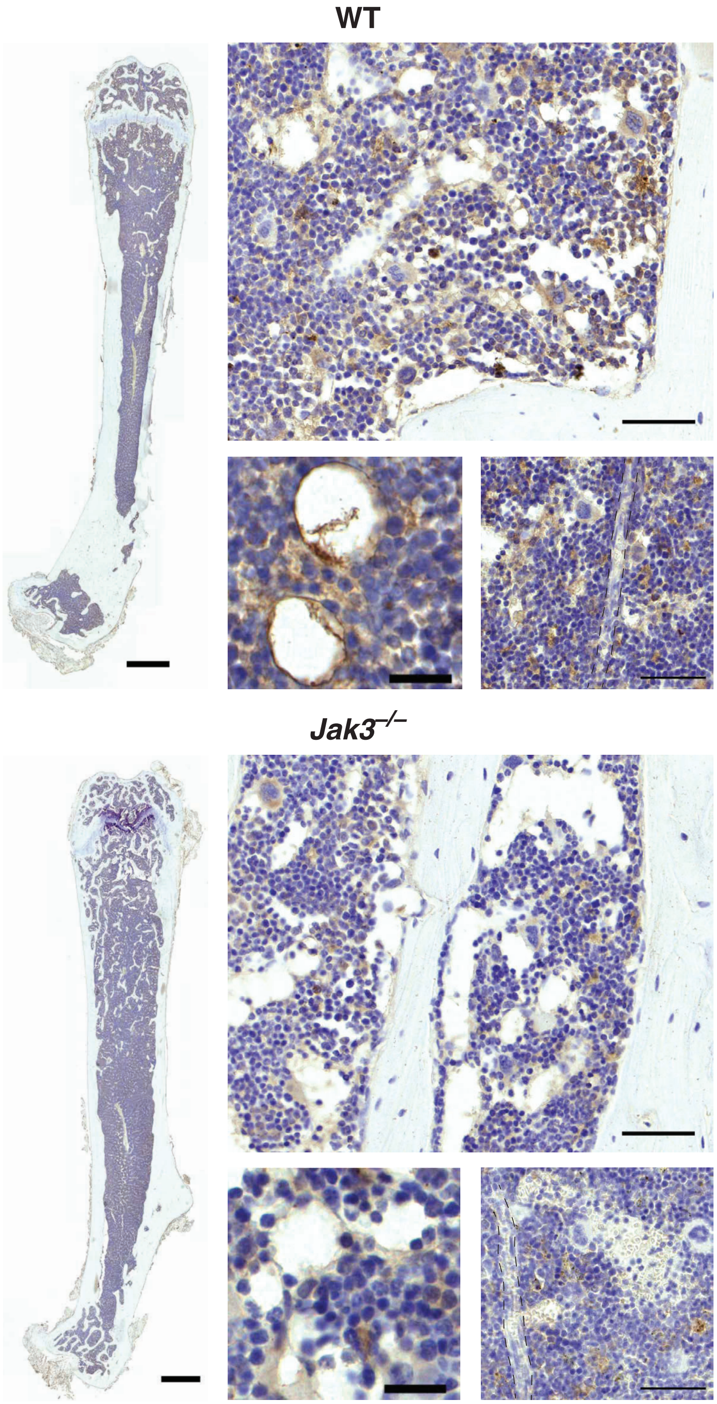

Supplementary Figure 2. Jak3 protein expression in WT and *Jak3*–/– mice.
Representative images of chromogen-based immunohistochemistry staining of WT (top) and *Jak3*–/– (bottom) femurs (n = 3 per group). Dashed line indicates trajectory of arteriole. Clockwise from top left, scale bars are 1 mm, 50 μm, 50 μm, and 20 μm for each group.

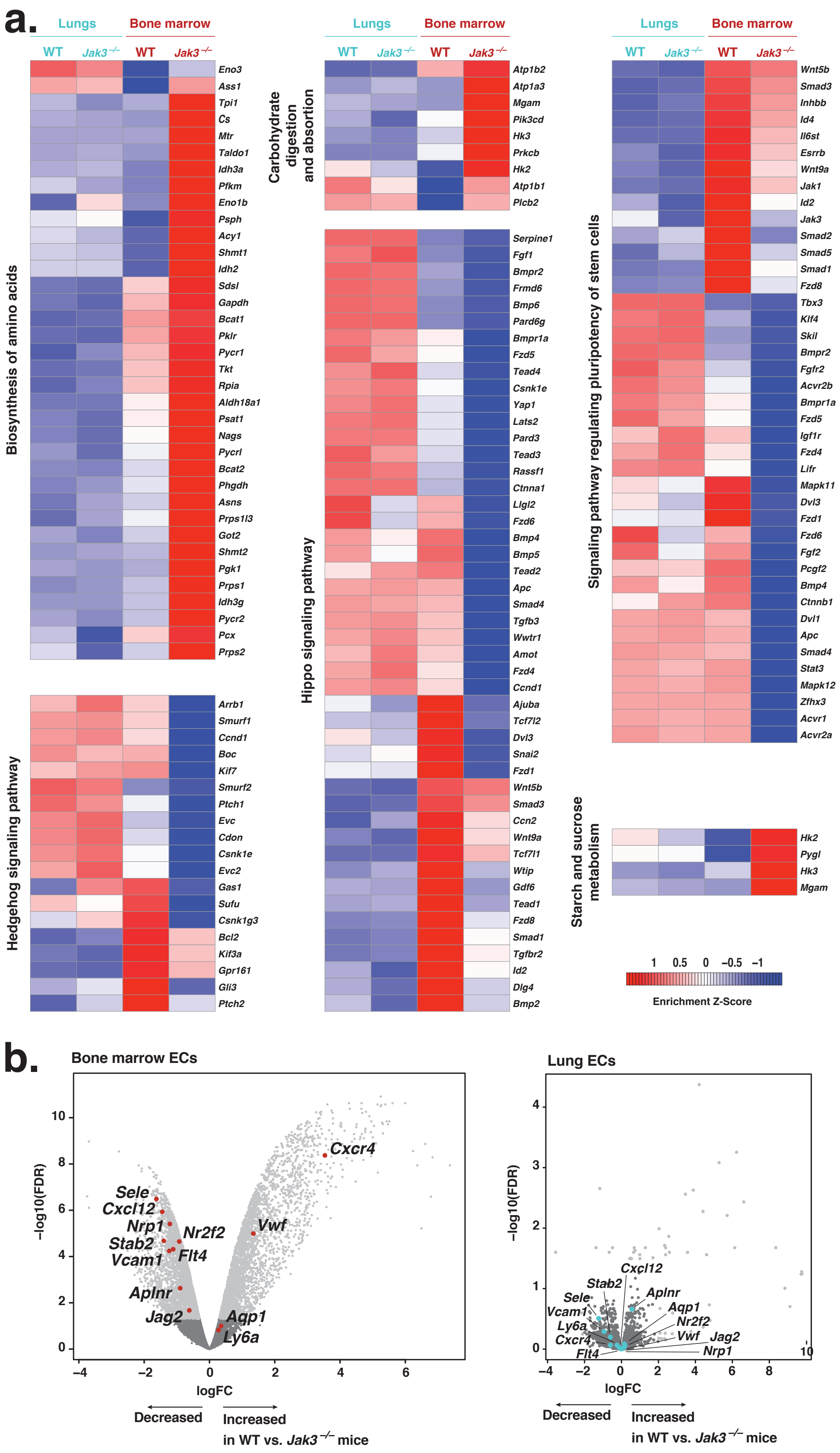

a.
Lungs
Bone marrow
WT
Jak3 −/−
Biosynthesis of amino acids
Eno3
Ass1
Tpi1
Cs
Mtr
Taldo1
Idh3a
Pfkm
Eno1b
Psph
Acy1
Shmt1
Idh2
Sdsl
Gapdh
Bcat1
Pklr
Pycr1
Tkt
Rpia
Aldh18a1
Psat1
Nags
Pycrl
Bcat2
Phgdh
Asns
Prps1l3
Got2
Shmt2
Pgk1
Prps1
Idh3g
Pycr2
Pcx
Prps2
Hedgehog signaling pathway
Arrb1
Smurf1
Ccnd1
Boc
Kif7
Smurf2
Ptch1
Evc
Cdon
Csnk1e
Evc2
Gas1
Sufu
Csnk1g3
Bcl2
Kif3a
Gpr161
Gli3
Ptch2
Carbohydrate digestion and absorption
Atp1b2
Atp1a3
Mgam
Pik3cd
Hk3
Prkcb
Hk2
Atp1b1
Plcb2
Hippo signaling pathway
Serpine1
Fgf1
Bmpr2
Frmd6
Bmp6
Pard6g
Bmpr1a
Fzd5
Tead4
Csnk1e
Yap1
Lats2
Pard3
Tead3
Rassf1
Ctnna1
Llgl2
Fzd6
Bmp4
Bmp5
Tead2
Apc
Smad4
Tgfb3
Wwtr1
Amot
Fzd4
Ccnd1
Ajuba
Tcf7l2
Dvl3
Snai2
Fzd1
Wnt5b
Smad3
Ccn2
Wnt9a
Tcf7l1
Wtip
Gdf6
Tead1
Fzd8
Smad1
Tgfbr2
Id2
Dlg4
Bmp2
Signaling pathway regulating pluripotency of stem cells
Wnt5b
Smad3
Inhbb
Id4
Il6st
Esrrb
Wnt9a
Jak1
Id2
Jak3
Smad2
Smad5
Smad1
Fzd8
Tbx3
Klf4
Skil
Bmpr2
Fgfr2
Acvr2b
Bmpr1a
Fzd5
Igf1r
Fzd4
Lifr
Mapk11
Dvl3
Fzd1
Fzd6
Fgf2
Pcgf2
Bmp4
Ctnnb1
Dvl1
Apc
Smad4
Stat3
Mapk12
Zfhx3
Acvr1
Acvr2a
Starch and sucrose metabolism
Hk2
Pygl
Hk3
Mgam
1 0.5 0 −0.5 −1
Enrichment Z-Score
b.
Bone marrow ECs
−log10(FDR)
logFC
Cxcr4
Sele
Cxcl12
Nrp1
Nr2f2
Vwf
Stab2
Flt4
Vcam1
Aplnr
Jag2
Aqp1
Ly6a
Decreased
Increased
in WT vs. Jak3 −/− mice
Lung ECs
−log10(FDR)
logFC
Cxcl12
Stab2
Aplnr
Sele
Vcam1
Aqp1
Nr2f2
Ly6a
Vwf
Jag2
Cxcr4
Nrp1
Flt4
Decreased
Increased
in WT vs. Jak3 −/− mice

Supplementary Figure 3. Lung and bone marrow endothelial cell (EC) signatures in WT and *Jak3*$^{-/-}$ mice. (**a**) Heatmaps depicting normalized transcript abundances of genes that contributed to the dysregulated Kyoto Encyclopedia of Genes and Genomes (KEGG)[54–56] terms generated by Gene Set Enrichment Analysis (GSEA) as depicted in Fig. 2c that are missing from Fig. 2d (n = 2 or 3 per organ). (**b**) Volcano plot illustrating differential expression analysis of WT vs. *Jak3*$^{-/-}$ bone marrow (top) and lung (bottom) ECs. Arterial and sinusoidal gene transcripts are highlighted with enlarged dots in red (bone marrow) and blue (lung); respectively, light or dark gray dots indicate gene transcripts differentially expressed above or below cutoffs, $-\log_{10}$(p value) < 1 and $|\log_2 FC| < 2.5$.

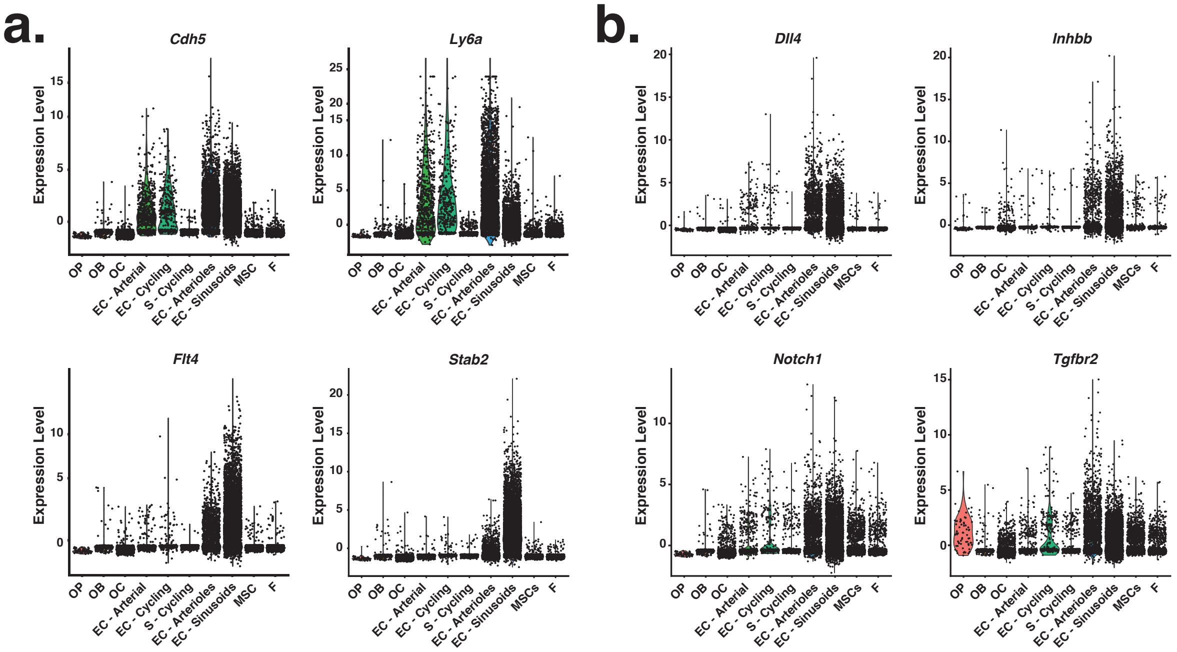

Supplementary Figure 4. Specific transcript expression in the bone marrow microenvironment at the single-cell level. (**a**) Violin plots illustrating canonical pan-endothelial (*Cdh5*), arterial (*Ly6a*), and sinusoidal (*Flt4* and *Stab2*) transcript abundances. (**b**) Violin plots illustrating Notch signaling pathway ligand (*Dll4*) and receptor (*Notch*) as well Tgfβ signaling pathway ligand (*Inhbb*) and receptor (*Tgfbr2*) transcript abundances. Data were obtained from combined single-cell RNA sequencing analysis of bone marrow cells from Tikhonova et al. (2019; GEO: GSE108892) and Baryawno et al. (2019; GEO: GSE128423). Each dot represents one cell.

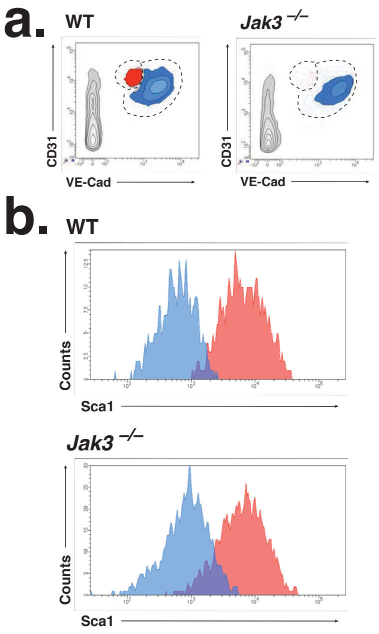

Supplementary Figure 5. Flow cytometry of primary WT and *Jak3*$^{-/-}$ bone marrow endothelial cells (ECs). (**a**) Representative flow cytometry plots showing bone marrow EC surface expression of canonical pan-endothelial markers CD31 and VE-Cad in WT (left) and *Jak3*$^{-/-}$ (right) mice (*n* = 10). (**b**) Representative flow cytometry histograms showing bone marrow EC surface expression of Sca1 in WT (top) and *Jak3*$^{-/-}$ (bottom) mice (*n* = 10).

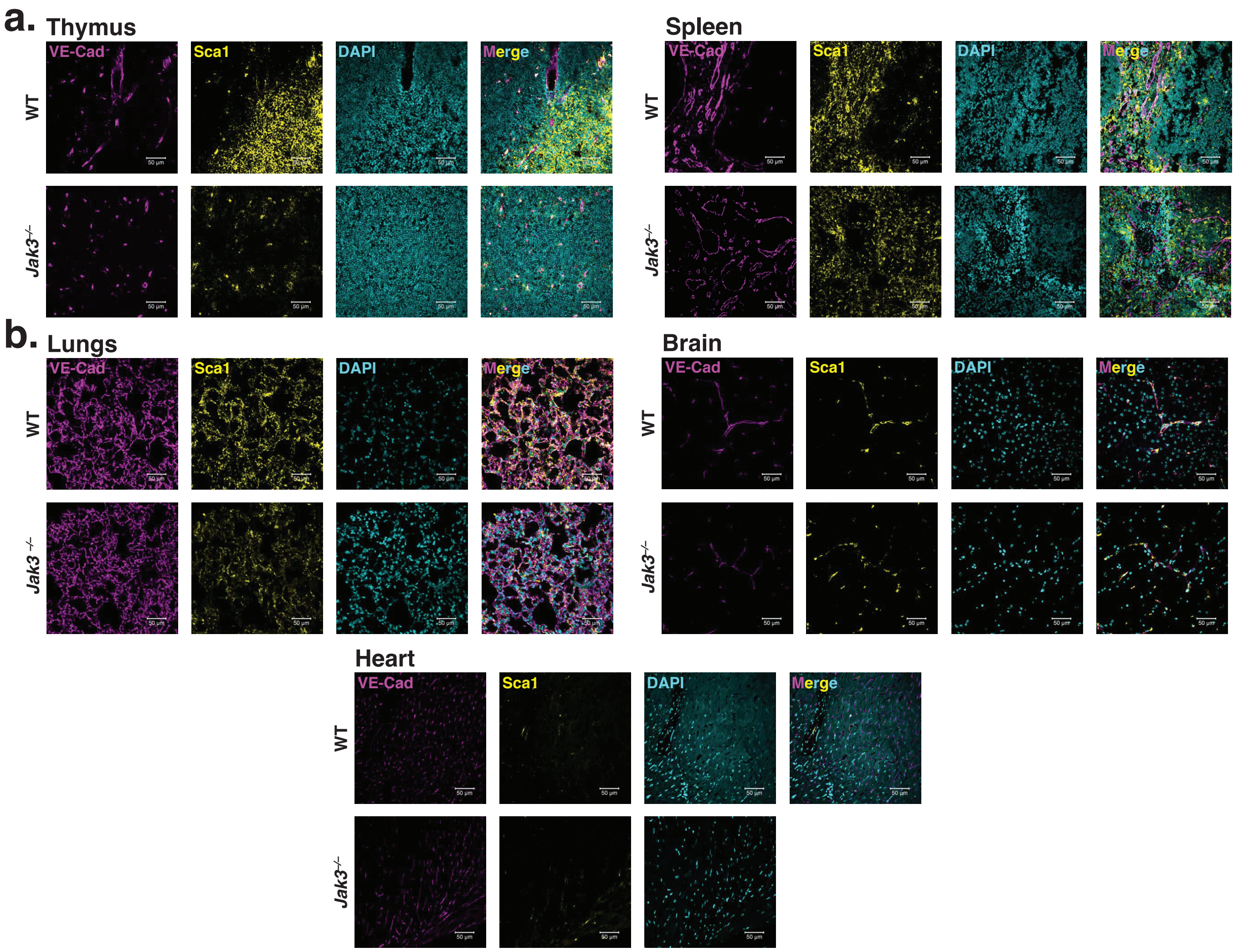

Supplementary Figure 6. Sca1 expression in various organs from WT and *Jak3*$^{-/-}$ mice. (**a**) Representative anti-VE-Cad (magenta), anti-Sca1 (yellow), and DAPI (cyan) immunofluorescent staining of WT (top) and *Jak3*$^{-/-}$ (bottom) of indicated secondary hematopoietic organs (n = 3). (**b**) Representative anti-VE-Cad (magenta), anti-Sca1 (yellow), and DAPI (cyan) immunofluorescent staining of WT (top) and *Jak3*$^{-/-}$ (bottom) of indicated non-hematopoietic organs (n = 3). Scale bars are 50 µm.

a.

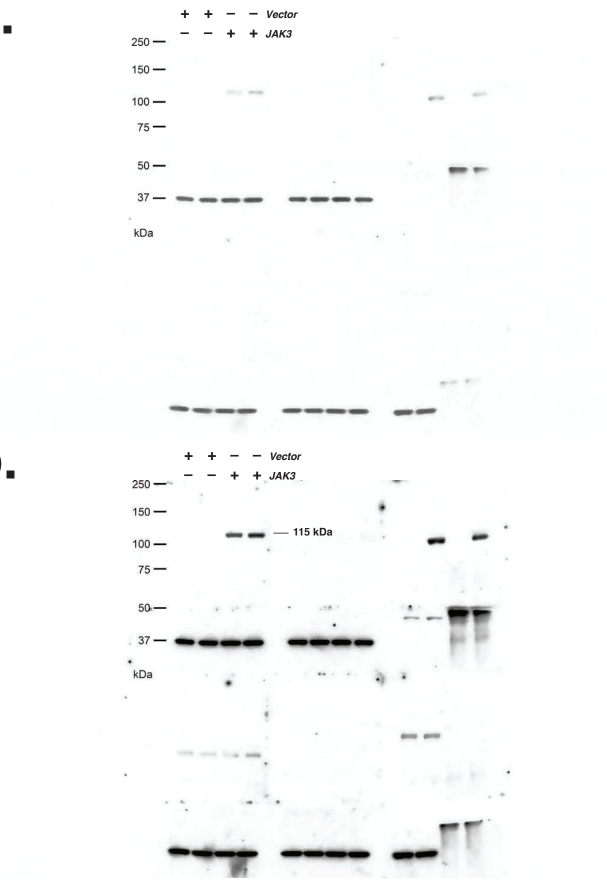

Supplementary Figure 7. Uncropped Western blots probing JAK3 protein in *JAK3*-transduced human ECs (ECs). (**a**) Full, uncropped Western blot membrane developed with exposure to observe GAPDH protein expression at 37 kDa. (**b**) Full, uncropped Western blot membrane developed with exposure to observe JAK3 protein expression at 115 kDa, as indicated on the right. The protein ladder used as reference has been recreated manually on the left. Both blots correspond to *Vector*- or *JAK3*-transduced ECs for in vitro co-culture experiments (n = 2). They have been cropped for display on Fig. 3a.

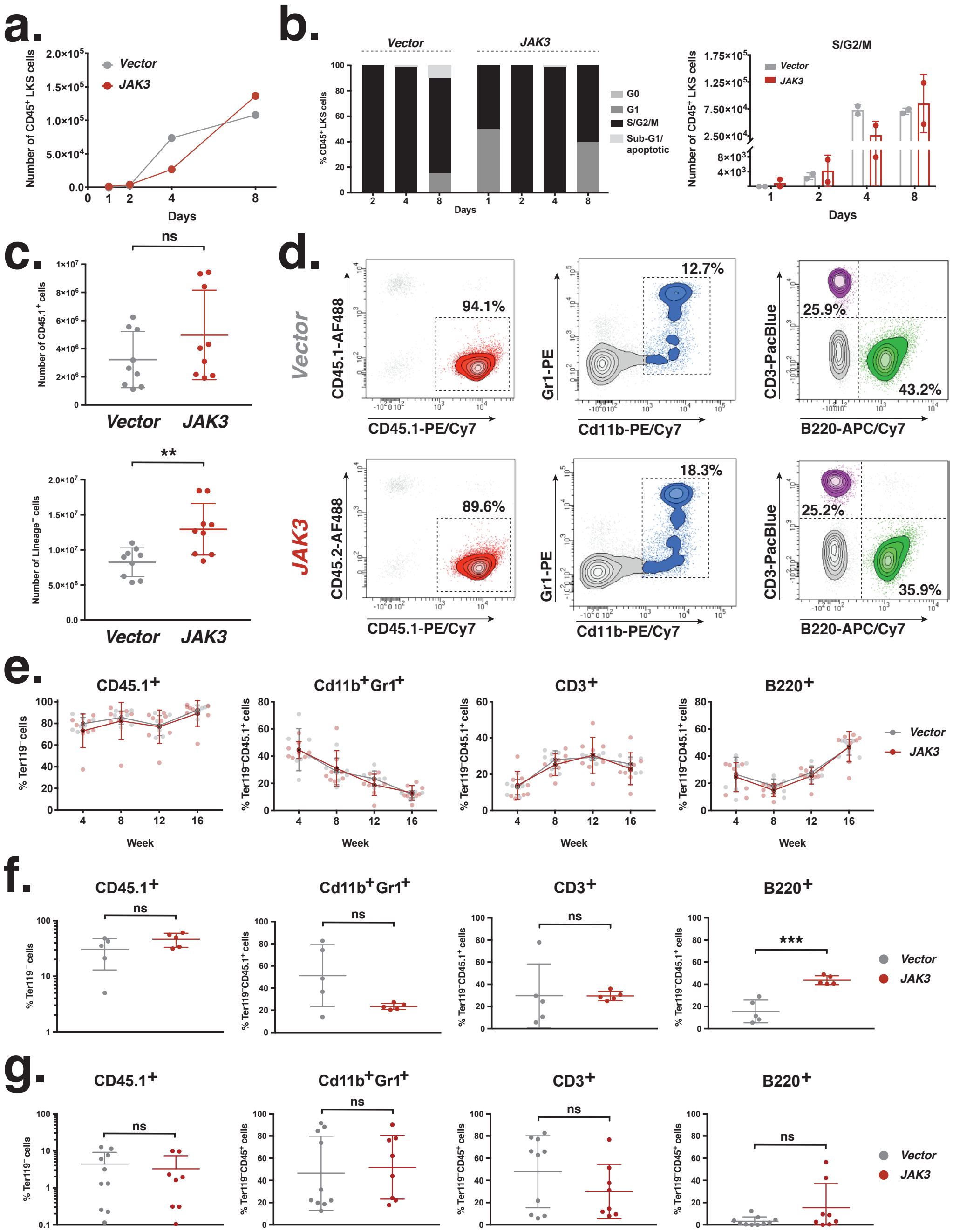

a.
Number of CD45+ LKS cells
Vector
JAK3
Days
b.
Vector
JAK3
% CD45+ LKS cells
G0
G1
S/G2/M
Sub-G1/ apoptotic
Days
S/G2/M
Number of CD45+ LKS cells
Days
c.
ns
Number of CD45.1+ cells
Vector
JAK3
**
Number of Lineage− cells
d.
Vector
CD45.1-AF488
CD45.1-PE/Cy7
94.1%
Gr1-PE
Cd11b-PE/Cy7
12.7%
CD3-PacBlue
B220-APC/Cy7
25.9%
43.2%
JAK3
CD45.2-AF488
89.6%
18.3%
25.2%
35.9%
e.
CD45.1+
Cd11b+Gr1+
CD3+
B220+
% Ter119− cells
% Ter119−CD45.1+ cells
Week
Vector
JAK3
f.
ns
***
g.
% Ter119−CD45+ cells

Supplementary Figure 8. Characterization of Lineage⁻cKit⁺Sca1⁺ (LKS) cell expansion on *Vector*- and *JAK3*-overexpressing human endothelial cells (ECs).
(**a**) Proliferation study quantifying the number of LKS cells in co-culture with *Vector*-(grey) or *JAK3*-(red) transduced ECs on days 1, 2, 4, and 8 of expansion ($n = 2$). (**b**) Cell cycle analysis of LKS cells in co-culture with Vector- (grey) or *JAK3*-(red) transduced endothelial cells (ECs) on days 1, 2, 4, and 8 of expansion (left) and total number of cycling (S/G2/M) LKS cells on each time point (right; $n = 2$). Each replicate is represented by an individual dot. (**c**) Measurement of CD45.1^{+} (top) and Lineage^{-} (bottom) cell expansion respectively gated on DAPI^{-} and DAPI^{-}CD45.1^{+} single cells by flow cytometry. Center lines represent the average number of cells. (**d**) Representative flow cytometry plots depicting peripheral blood (PB) chimerism (left, red) as well as myeloid (center, blue), T lymphoid (right, purple), and B lymphoid (right, green) staining 16 weeks following transplantation of LKS cells into lethally irradiated CD45.2^{+} recipients after co-culture with *Vector*- (top) or *JAK3*- (bottom) transduced ECs ($n = 10$ per group). (**e**) Quantification of monthly flow cytometry analyses of chimerism and lineage contribution to the PB over the course of 4 months following competitive transplantation. From left to right: donor chimerism, myeloid cells, T lymphoid cells, and B lymphoid cells. (**f**) Chimerism and lineage distribution of PB from murine cohort that received highest cell dose 20 weeks after limiting-dilution transplantation of LKS cells following co-culture with *Vector*- (grey) or *JAK3*-(red) transduced ECs ($n = 10$ per group). From left to right: donor chimerism, myeloid cells, T lymphoid cells, and B lymphoid cells. Center lines represent the average number of cells. (**g**) Chimerism and lineage distribution of PB from secondary-transplanted mice that received whole bone marrow from highest-dose, primary-transplanted experimental (*Vector,* grey) and control (*JAK3,* red) groups 16 weeks after limiting-dilution transplantation. From left to right: donor chimerism, myeloid cells, T lymphoid cells, and B lymphoid cells. Center lines represent the average number of cells. Three asterisks (***), two asterisks (**), and ns (not significant) denote, respectively, p values < 0.001, < 0.01, and > 0.05 by unpaired, two-tailed Student's t test. On all plots, each replicate is represented by an individual dot. On line plots, mean values are joined by a connecting line and every replicate is depicted in a lighter shade of its corresponding color. All error bars represent the standard deviation from the mean.

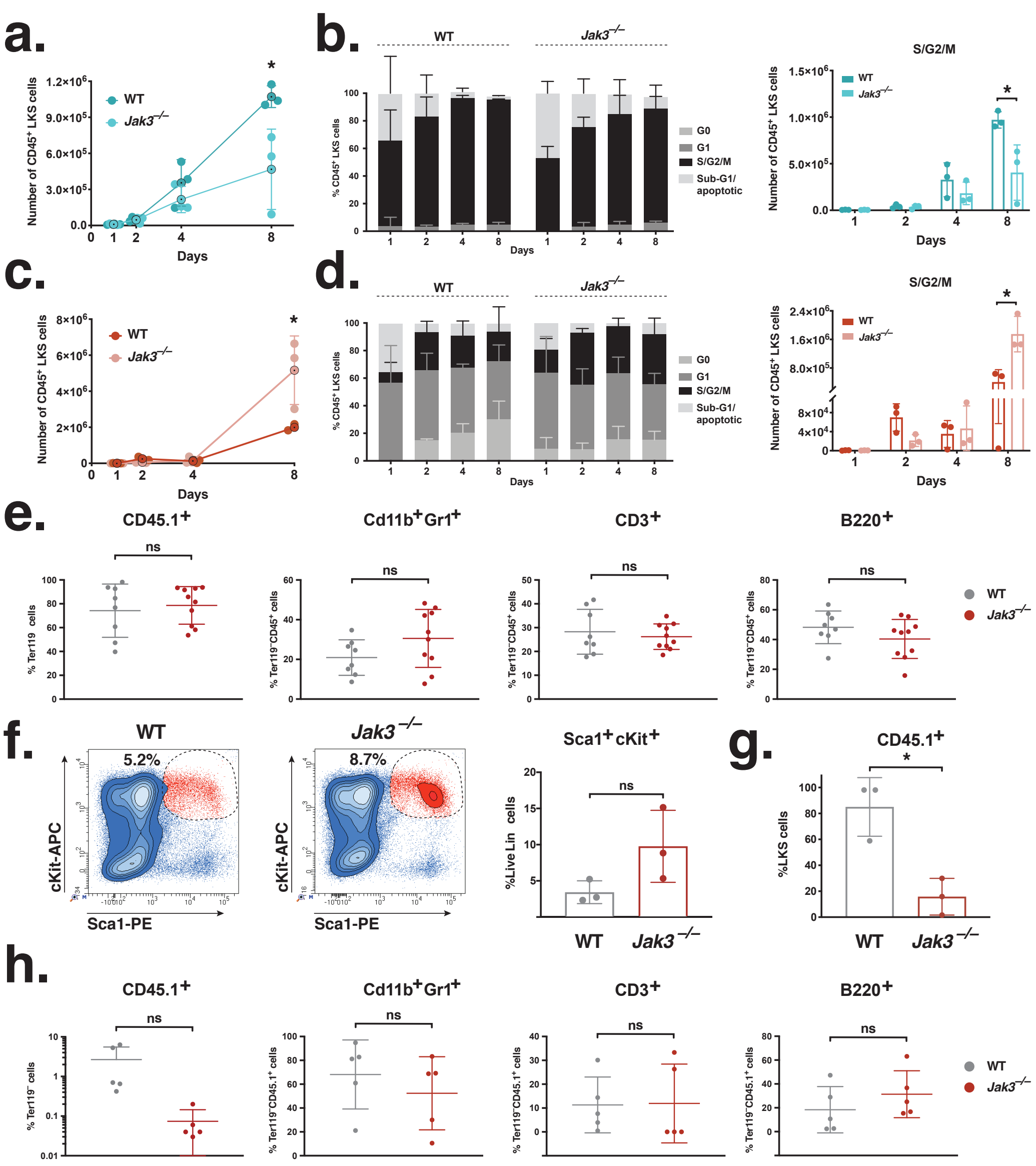

a.
b.
c.
d.
e.
f.
g.
h.
WT
Jak3−/−
Number of CD45+ LKS cells
Days
% CD45+ LKS cells
G0
G1
S/G2/M
Sub-G1/ apoptotic
S/G2/M
CD45.1+
Cd11b+Gr1+
CD3+
B220+
ns
% Ter119 cells
% Ter119−CD45+ cells
cKit-APC
Sca1-PE
5.2%
8.7%
Sca1+cKit+
%Live Lin cells
%LKS cells
% Ter119− cells
% Ter119−CD45.1+ cells

Supplementary Figure 9. Characterization of Lineage$^-$cKit$^+$Sca1$^+$ (LKS) cell expansion on WT and *Jak3$^{-/-}$* lung and bone marrow endothelial cells (ECs).

(**a**) Proliferation study quantifying the number of LKS cells in co-culture with WT (dark blue) or *Jak3$^{-/-}$* (light blue) murine lung ECs on days 1, 2, 4, and 8 of expansion (*n* = 3). (**b**) Cell cycle analysis of LKS cells in co-culture with WT or *Jak3$^{-/-}$* murine lung ECs on days 1, 2, 4, and 8 of expansion (left) and total number of cycling (S/G2/M) LKS cells on each time point (right; *n* = 3). (**c**) Proliferation study quantifying the number of Lineage$^-$cKit$^+$Sca1$^+$ (LKS) cells in co-culture with WT (dark red) or *Jak3$^{-/-}$* (light red) murine bone marrow ECs on days 1, 2, 4, and 8 of expansion (*n* = 3). (**d**) Cell cycle analysis of LKS cells in co-culture with WT or *Jak3$^{-/-}$* murine bone marrow ECs on days 1, 2, 4, and 8 of expansion (left) and total number of cycling (S/G2/M) LKS cells on each time point (right; *n* = 3). (**e**) Chimerism and lineage distribution of peripheral blood (PB) 20 weeks following primary transplantation into WT (grey) or *Jak3$^{-/-}$* (red) mice, prior to limiting-dilution secondary transplantation (*n* = 8 and 10, respectively). From left to right: donor chimerism, myeloid cells, T lymphoid cells, and B lymphoid cells. Center lines represent the average number of cells. (**f**) Representative flow cytometry plots depicting bone marrow-derived LKS cells from primary-transplanted mice 20 weeks following primary transplantation into WT (grey) or *Jak3$^{-/-}$* (red) mice, prior to limiting-dilution secondary transplantation (left and center; *n* = 3 per group) and averaged LKS cell percentages (right). (**g**) Averaged percent chimerism of LKS sub-population 20 weeks following primary transplantation into WT (grey) or *Jak3$^{-/-}$* (red) mice (*n* = 3 per group). (h) Chimerism and lineage distribution of PB from murine cohort that received highest cell dose 20 weeks after secondary transplantation of LKS in a limiting-dilution manner (*n* = 5 per group), including specimens that did not meet the threshold for engraftment (0.2% and higher percent chimerism). From left to right: donor chimerism, myeloid cells, T lymphoid cells, and B lymphoid cells. Center lines represent the average number of cells. One asterisk (*) and ns (not significant) denote, respectively, *p* values < 0.05 and > 0.05 by unpaired, two-tailed Student's *t* test. On all plots, each replicate is represented by an individual dot. On line plots, mean values are joined by a connecting line and every replicate is depicted in a lighter shade of its corresponding color. All error bars represent the standard deviation from the mean.
