# Supplementary material for: Endothelial Jak3 expression enhances pro-hematopoietic angiocrine function in mice
Source: Commun Biol. 2021 Mar 25;4:406. doi: 10.1038/s42003-021-01846-3 (PMC7994450; doi:10.1038/s42003-021-01846-3)
Supplement: Supplementary file 2 — Description of Additional Supplementary Files [file 42003_2021_1846_MOESM2_ESM.pdf]

Description of Additional Supplementary Files

File name: Supplementary Data 1

Description: Bulk RNA sequencing data for WT endothelial cells in various tissues.

File name: Supplementary Data 2

Description: Bulk RNA sequencing data for *Jak3* KO and WT endothelial cells in lung and bone marrow.

File name: Supplementary Data 3

Description: Source data for Figures 2d, 3, and 4; and Supplementary Figures 1d, 8, and 9.
