# Supplementary material for: Endothelial Jak3 expression enhances pro-hematopoietic angiocrine function in mice
Source: Commun Biol. 2021 Mar 25;4:406. doi: 10.1038/s42003-021-01846-3 (PMC7994450; doi:10.1038/s42003-021-01846-3)
Supplement: Supplementary file 6 — Reporting Summary [file 42003_2021_1846_MOESM6_ESM.pdf]

nature research

Corresponding author(s): David Redmond, Raphael Lis

Last updated by author(s): Jan 26, 2020

Reporting Summary

Nature Research wishes to improve the reproducibility of the work that we publish. This form provides structure for consistency and transparency in reporting. For further information on Nature Research policies, see our Editorial Policies and the Editorial Policy Checklist.

Statistics

For all statistical analyses, confirm that the following items are present in the figure legend, table legend, main text, or Methods section.

n/a	Confirmed	
☐	☒	The exact sample size (*n*) for each experimental group/condition, given as a discrete number and unit of measurement
☐	☒	A statement on whether measurements were taken from distinct samples or whether the same sample was measured repeatedly
☐	☒	The statistical test(s) used AND whether they are one- or two-sided<br>*Only common tests should be described solely by name; describe more complex techniques in the Methods section.*
☐	☒	A description of all covariates tested
☐	☒	A description of any assumptions or corrections, such as tests of normality and adjustment for multiple comparisons
☐	☒	A full description of the statistical parameters including central tendency (e.g. means) or other basic estimates (e.g. regression coefficient) AND variation (e.g. standard deviation) or associated estimates of uncertainty (e.g. confidence intervals)
☐	☒	For null hypothesis testing, the test statistic (e.g. *F*, *t*, *r*) with confidence intervals, effect sizes, degrees of freedom and *P* value noted<br>*Give P values as exact values whenever suitable.*
☒	☐	For Bayesian analysis, information on the choice of priors and Markov chain Monte Carlo settings
☒	☐	For hierarchical and complex designs, identification of the appropriate level for tests and full reporting of outcomes
☒	☐	Estimates of effect sizes (e.g. Cohen's *d*, Pearson's *r*), indicating how they were calculated

Our web collection on statistics for biologists contains articles on many of the points above.

Software and code

Policy information about availability of computer code

Data collection	Sequencing data was generation using an Illumina HiSeq 4000.
Data analysis	Raw sequencing data was quality-controlled and analyzed using FastQC (v0.11.5), DEE2, Skewer (v0.2.2), Minion (Kraken package), the STAR aligner, edgeR, WebGestalt, the STRING app on Cytoscape (v3.8.0.83), and Graphpad Prism (v7.0d). Single cell data was processed using the Seurat package (v3.1.4) on R (v3.6.1).

For manuscripts utilizing custom algorithms or software that are central to the research but not yet described in published literature, software must be made available to editors and reviewers. We strongly encourage code deposition in a community repository (e.g. GitHub). See the Nature Research guidelines for submitting code & software for further information.

Data

Policy information about availability of data

All manuscripts must include a data availability statement. This statement should provide the following information, where applicable:

- Accession codes, unique identifiers, or web links for publicly available datasets
- A list of figures that have associated raw data
- A description of any restrictions on data availability

All original bulk cell expression matrices have been deposited in the Gene Expression Omnibus (GSE159755)

Field-specific reporting

Please select the one below that is the best fit for your research. If you are not sure, read the appropriate sections before making your selection.

☒ Life sciences ☐ Behavioural & social sciences ☐ Ecological, evolutionary & environmental sciences

For a reference copy of the document with all sections, see nature.com/documents/nr-reporting-summary-flat.pdf

Life sciences study design

All studies must disclose on these points even when the disclosure is negative.

Sample size	No sample size calculations were performed for this study.
Data exclusions	Negative data was excluded.
Replication	Generally, results were found consistent in 3 or more biological replicates. High-throughout experiments were found consistent 2 or more biological replicates.
Randomization	Mouse littermates were from experimental and control groups were sex-matched.
Blinding	Mice had to be sorted by genotype.

Reporting for specific materials, systems and methods

We require information from authors about some types of materials, experimental systems and methods used in many studies. Here, indicate whether each material, system or method listed is relevant to your study. If you are not sure if a list item applies to your research, read the appropriate section before selecting a response.

Materials & experimental systems

n/a	Involved in the study	
☐	☒	Antibodies
☐	☒	Eukaryotic cell lines
☒	☐	Palaeontology and archaeology
☐	☒	Animals and other organisms
☒	☐	Human research participants
☒	☐	Clinical data
☒	☐	Dual use research of concern

Methods

n/a	Involved in the study	
☒	☐	ChIP-seq
☐	☒	Flow cytometry
☒	☐	MRI-based neuroimaging

Antibodies

Antibodies used

Antibody, fluorophore(s), clone(s), catalog number(s):
VE-Cadherin, AF647, BV13, 138006
CD31, PE/Cy7, 390, 102418
CD45, PE, 30-F11, 103106, 103116
c-Kit, APC, 2B8, 105812
Sca1, (PE, PE/Cy7), D7, (108108, 108114)
Lineage, (BV421/FITC), (combination 145-2c11, RB6-8C5, RA3-6B2, Ter-119, and M1/70), (133311, 133302)
Ter119, (BV421, APC), Ter119, (116233, 116212)
CD45.2, (AF488, APC/Cy7, AF700), 104, (109816, 109824, 109822)
CD45.1, PE/Cy7, A20, 110730
CD3, BV421, 17A2, 100228
B220, (APC/Cy7, BV421), RA3-6B2, (103224, 103240)
Gr1, (PE, FITC), RB6-8C5, (108408, 108406)
CD11b (PE, PE/Cy7, AF700) M1/70 (101208, 101216, 101222)
All from Biolegend.

Validation

Validation is available on the manufacturers' websites.

Eukaryotic cell lines

Policy information about cell lines

Cell line source(s)	Mouse cells were isolated from the lungs and bone marrow of experimental and control groups. Human umbilical vein endothelial cells were isolated from umbilical cords. No commercial lines were used.
Authentication	The cell lines were not authenticated.
Mycoplasma contamination	The cell lines were not tested for mycoplasma.
Commonly misidentified lines (See ICLAC register)	No commonly misidentified cell lines were used in this study.

Animals and other organisms

Policy information about studies involving animals; ARRIVE guidelines recommended for reporting animal research

Laboratory animals	We used wildtype (WT) mice (C57BL/6J; Jackson Laboratory, strain 000664) for RNA-sequencing or in vitro culture. For loss-of-function experiments, we used a knock-in mouse model (B6;129S4-Jak3tm1Ljb/J; Jackson Laboratory, strain 002852). For transplantation experiments, we used mice that express the CD45.1 isoform of the Ptprc gene only (B6.SJL-Ptprca Pepcb/BoyJ; Jackson Laboratory, strain 002014). All animal experiments included at least 5 animals per group, and all groups included a litter-dependent, near-balanced ratio of males to females. All animals used were 10–12 weeks of age and age- and sex-matched between control and experimental groups when applicable. No animals were excluded from quantification. We analyzed scRNA-sequencing data obtained from bone marrow cells from Col1a1-creERT2;tdTomatolox/stop/lox, Lepr-cre;tdTomatolox/stop/lox, Cdh5-cre;tdTomatolox/stop/lox, as well as C57BL/6J mice (see Tikhonova et al., 2019, and Baryawno et al., 2019).
Wild animals	The study did not involve wild animals.
Field-collected samples	The study did not involve samples collected from the field.
Ethics oversight	All animal manipulation was carried out with the approval of Weill Cornell Medicine Institutional Animal Care and Use Committee.

Note that full information on the approval of the study protocol must also be provided in the manuscript.

Flow Cytometry

Plots

Confirm that:

- [x] The axis labels state the marker and fluorochrome used (e.g. CD4-FITC).
- [x] The axis scales are clearly visible. Include numbers along axes only for bottom left plot of group (a 'group' is an analysis of identical markers).
- [x] All plots are contour plots with outliers or pseudocolor plots.
- [x] A numerical value for number of cells or percentage (with statistics) is provided.

Methodology

Sample preparation	For fluorescent activated cell sorting (FACS) for bulk RNA-sequencing, the livers, lungs, kidneys, hearts, and bones of individual mice were harvested 15 min after retro-orbital injection of 25 µg anti-VE-Cadherin-AF647. Soft tissues were were minced and placed in 25 mg ml−1 Collagenase A, 1 unit per ml Dispase II , and 50 µg ml−1 DNase I in Hank's Balanced Salt Solution at 37 °C for 20–30 min to create a single-cell suspension. For bone marrow isolation, the sternum, pelvic bones, femurs, and tibias were mechanically denuded of muscle and connective tissue, crushed using a mortar and pestle, and placed in the same digestion solution as above and at the same temperature for 15 min. All tissue digests were filtered through a 40-µm sieve and centrifuged at 500 g for 10 min and co-stained using fluorochrome-conjugated antibodies against Ter119, CD45, and CD31. For FACS for hematopoietic stem and progenitor cell expansion in vitro, bones were not enzymatically digested. Instead, Lineage-committed hematopoietic cells were depleted by magnetic separation using microbeads conjugated to monoclonal antibodies against CD5, B220, CD11b, Gr-1, and Ter-119 and co-stained using fluorochrome-conjugated antibodies against CD45, Sca1, cKit, and a Lineage cocktail. For FACS for transplantation of expanded hematopoietic cells, whole co-cultures were enzymatically digested using Accutase and co-stained using fluorochrome-conjugated antibodies against CD45, Sca1, cKit, and a Lineage cocktail. For flow cytometric analyses of peripheral blood, 35 µl blood were retrieved retro-orbitally from anesthetized transplant recipient mice using heparinized micro-hematocrit capillary tubes. Red blood cells were depleted and the remaining cell suspensions were washed twice at 300 g for 10 min in PBS containing 2 mM EDTA and 0.2% wt/vol bovine serum albumin. Cell suspensions were co-stained for using fluorochrome-conjugated antibodies against Ter119, CD45.2, and CD45.1. To prevent non-specific antibody binding, cell pellets from all experiments were re-suspended in a 1:50 solution of FcR Blocking Reagent in PBS containing 2 mM EDTA and 0.2% wt/vol bovine serum albumin for 10 min at 4 °C prior to co-staining. Blocked cell suspensions were co-stained for 30 min at 4 °C at a concentration of 0.2 µg per 1 million cells. Prior to sorting, stained samples were washed once and resuspended in blocking buffer with 1 µg ml−1 DAPI for viability discrimination.
Instrument	BD FACSAria II

Software	BD FACSDiva 8.0.1
Cell population abundance	NA
Gating strategy	All single-cell suspensions were gated for size and morphology, then viability (DAPI-negative). Endothelial cells were gated as Ter119-negative, CD45-negative, CD31-positive, and VECad-positive. Hematopoietic stem and progenitor cells for transplantation were gated as Ter119-negative, CD45-negative, Lineage-negative, cKit-positive, and Sca1-positive. Chimerism of peripheral blood cells was gated as Ter119-negative, CD45.2-negative, CD45.1-positive. Peripheral blood myeloid cells were gated as Ter119-negative, CD45-positive, Gr1-positive, CD11b-positive; T lymphoid cells as Ter119-negative, CD45-positive, CD3-positive; and B lymphoid cells as Ter119-negative, CD45-positive, B220-positive.

☒ Tick this box to confirm that a figure exemplifying the gating strategy is provided in the Supplementary Information.
